# Rehabilitation utilisation up to 24 months after stroke: a register-based cohort study in southern Sweden

**DOI:** 10.1093/esj/aakag086

**Published:** 2026-07-24

**Authors:** Martin Ringsten, Susanne Iwarsson, Bo Norrving, Eva Månsson Lexell

**Affiliations:** Department of Health Sciences, Lund University, Lund, Sweden; Department of Health Sciences, Lund University, Lund, Sweden; Department of Clinical Sciences, Neurology, Lund University, Lund, Sweden; Department of Health Sciences, Lund University, Lund, Sweden; Department of Neurology, Rehabilitation Medicine, Memory Disorders, and Geriatrics, Skåne University Hospital, Lund-Malmö, Sweden

**Keywords:** delivery, healthcare utilisation, occupational therapy, physiotherapy, rehabilitation, stroke

## Abstract

**Introduction:**

Knowledge about the quantity, delivery and setting of rehabilitation after stroke in clinical practice is sparse. We investigated the utilisation of rehabilitation services in a regional cohort up to 2 years after stroke.

**Patients and methods:**

Data on people living in ordinary housing in southern Sweden with a first-ever stroke during 2016-2017 were collected from the Swedish Stroke Registry and linked to healthcare data from regional and private primary care settings, inpatient and outpatient hospital care and general population registries up to 24 months after stroke onset. Rehabilitation consultations after discharge were analysed using descriptive statistics, categorised by setting and delivery and stratified by age, living situation and sex.

**Results:**

A total of 3034 people were included, who received more than 41,000 rehabilitation consultations over 24 months. During the first 3 months 42% received rehabilitation, as did 42% during months 4–12 and 32% during months 13–24. People under 65 years received rehabilitation more often than people aged 65–74, 75–84 or older than 84 years (57%, 44%, 36% and 19%, respectively) during the initial 3 months. These differences persisted at 4–12 and 13–24 months. Over 24 months, 40% of the consultations were delivered in primary care, 28% in specialised outpatient hospital care and 32% in specialised inpatient hospital care. Furthermore, 55%, 38% and 7% of all consultations were delivered by multi-professional teams, physiotherapists and occupational therapists.

**Discussion:**

Less than half of the participants after stroke utilised rehabilitation during the 2 years and the intensity, setting and delivery varied over time. Further studies are warranted to evaluate if the amount of rehabilitation matches individual needs after stroke.

## Graphical Abstract

**Graphical Abstract:**
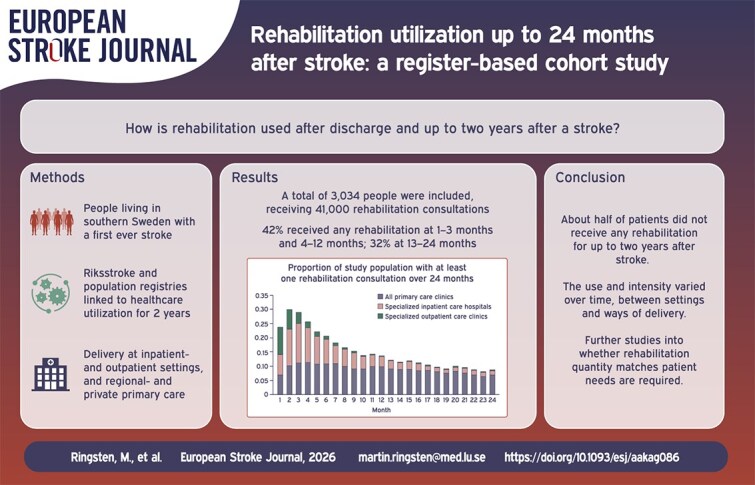

## Introduction

Each year around 25,000 people in Sweden sustain a stroke^1^ and approximately 1.1 million do so in Europe,^2^ making it one of the leading causes of disability worldwide.^3^ Perceived needs due to disability after a stroke are common^4^ but can differ depending on the level of disability and stage in the recovery process. These needs can be categorised as disease-related information needs; physical recovery, activity and participation needs; social environmental resources and psycho-emotional support needs.^5^ Unmet needs can be a target for healthcare services, where rehabilitation plays a central part.

Rehabilitation is defined by the WHO as “a set of interventions designed to optimise functioning and reduce disability in individuals with health conditions in interaction with their environment.”^6^ Rehabilitation, in Sweden and beyond, can be delivered by single healthcare professionals, commonly physiotherapists or occupational therapists, or by multi-professional teams including at least 1 physiotherapist or occupational therapist. Rehabilitation is delivered in different settings, such as inpatient or outpatient clinics in hospitals, and within primary care. Delivery, indications^7^ and the setting of rehabilitation differ within Sweden^8^ and across countries.^9^ These differences can be related to the structure of the healthcare system,^10^ available resources, perceived needs, level of disability and individual characteristics. A common experience after a stroke is uncertainty about where to receive support within the healthcare system to deal with disability and any unmet needs, especially in the longer term.^11^ The Stroke Action Plan for Europe^9^ sets the target that all people after stroke should have their rehabilitation needs reviewed after 3 months, and a majority should have a plan for rehabilitation outside the hospital if needed. Both Swedish^12^ and international guidelines^13^ state that rehabilitation should be continuous, including initial rehabilitation at a hospital stroke unit as well as long-term rehabilitation. Whether these recommendations are met warrants further investigation.

A recent scoping review explored the current evidence related to the extent and utilisation of stroke rehabilitation interventions.^14^ It identified that actual utilisation differed greatly between studies and only 1 previous study^15^ assessed rehabilitation utilisation more than 1 year after a stroke. This highlights the knowledge gap regarding the current utilisation of rehabilitation services. That is, where they are utilised, and who they are delivered by.

The aim of this study was to describe the utilisation of rehabilitation after discharge and up to 2 years after a first-ever stroke, identifying the settings in which rehabilitation was delivered and who delivered it. Furthermore, we investigated differences in rehabilitation utilisation considering age, sex and living situation.

## Methods

### Design

A cohort study design was used analysing data collected from several Swedish registries from 2016 to 2020. Individual level data were collected from several registry sources and linked based on the personal identification numbers of people who had a first-ever stroke.

The study is reported according to the Strengthening the Reporting of Observational Studies in Epidemiology (STROBE) statement.^16^ Ethics approval was granted by the Swedish Ethical Review Authority (no. 2024-00180-01).

### Study population, data sources and procedure

The study population was identified from the Swedish Stroke Registry^17^ and included community-dwelling people living in their own homes in Skåne County, who experienced a first-ever stroke event—including both ischaemic and haemorrhagic stroke—from January 2016 to December 2017. People with a transient ischaemic attack were excluded. Participants who died or moved away from the county during follow-up were identified using the Swedish National Population Register.^18^ Characteristics related to living situation, sex and age were collected from the Swedish Stroke Registry and data on demographics, income and educational levels were collected from the Longitudinal Integrated Database for Health Insurance and Labour Market Studies (LISA) and linked by Statistics Sweden. Healthcare utilisation from the stroke-event up to 2 years was collected from the Skåne Healthcare Registry,^19^ which includes hospital-based care and primary care provided by regional and private clinics. Primary health care provided by municipalities is not included. The registries were linked by Clinical Studies Sweden, using standard pseudonymisation procedures. Detailed descriptions of the registries, data collection and variables are available in the Supplementary material.

### Categorisation of rehabilitation consultations

A rehabilitation consultation was identified as a physical or digital meeting, delivered as an individual or group consultation within a clinic, hospital or at home, conducted after discharge from acute care, based on the medical record information included in the registry. *The delivery of consultations* was split into the following categories:

- Consultations delivered by a single rehabilitation professional (physiotherapist or occupational therapist): Consultations by both professions within the same day were analysed as separate consultations. Individual consultations by speech-language therapists, psychologists and other professions were not included in this study.
- Consultations delivered by a multi-professional team: Defined as including at least 2 or more professionals, and at least 1 physiotherapist/occupational therapist.

We placed no restrictions on the composition of the consultations. Thus, consultations could include assessments, interventions or follow-up visits. Consultations over the telephone or via letters were not included. At the time of the study, Early Supported Discharge was planned for approximately 4% of all people admitted to a stroke unit within the hospital region in this study.^8^ Because we utilised existing registry and healthcare visit data, no information about the content of the consultations, the clinician’s experience in stroke rehabilitation or the collaboration between the rehabilitation professionals involved was available. The intensity of rehabilitation was categorised into 4 groups based on the number of consultations (0; 1–2; 3–5; > 5).

*The setting of consultations* was categorised according to the structure of the Swedish healthcare system,^20^ and the medical records that the registry is based on:

- Primary care clinics, including all regional or privately run clinics that deliver rehabilitation. Registry data on consultations delivered by municipal primary care were not available.
- Outpatient specialised hospital-based care, including all outpatient clinics offering rehabilitation after stroke or general rehabilitation (eg, geriatric rehabilitation). Settings that were not primarily aimed at disability after stroke, but rather at other symptoms and diagnoses were excluded.
- Thirdly, inpatient specialised hospital-based care: Including all hospital-based clinics and wards offering rehabilitation after stroke. Wards that only handled people after stroke in the acute setting were not included. For inpatient specialised care, the registry data are based on days in the hospital with a registered meeting with healthcare personnel and were used in this study instead of individual consultation counts.

### Statistical analyses

Descriptive statistics were used to present baseline data. Results are presented for the proportion of people after stroke who received rehabilitation consultations and for the total number of consultations, separately. The comorbidity burden was calculated using the Charlson Comorbidity Index.^21^ Age groups were categories into < 65 years, 65–74, 75–84 and > 85 years of age at baseline. Chi-squared tests were used to investigate differences between groups and a 2-sided *P* value of < .05 was considered statistically significant. Stata version 18.0 was used for statistical analyses. Individuals who passed away or moved from Skåne County before the end of each month were not included in the utilisation data for the following months (see Supplementary material Table S1 for details).

## Results

A total of 3969 people had a first-ever stroke over the study period. Of those, 3034 lived in ordinary housing and a total of 41,224 rehabilitation consultations were recorded over 2 years after discharge for this population.

### Baseline characteristics

The study population had a mean age of 71.4 years (SD 12.3) and 65% had Charlson Comorbidity Index of 1 or more. The vast majority (88%) were fully conscious when arriving at the hospital. Study population characteristics are available in Table 1. Total population numbers included for each month, accounting for deaths and moves outside of the study district is available in the Supplementary material.

**Table 1:** Study population characteristics.

Characteristic	Overall (*n*: 3034)
Age, mean (SD) years	71.4 (12.3)
Men *n* (%)	1728 (57%)
Fully conscious at admittance to hospital *n* (%)	2640 (88%)^a^
Living alone before stroke *n* (%)	1136 (38%)^b^
Any support needs before stroke *n* (%)	219 (7%)^c^
Mobility limitations before stroke *n* (%)	61 (2%)^d^
CCI *n* (%)	
CCI 0 (%)	1076 (35%)
CCI 1–2 (%)	1034 (34%)
CCI 3–4 (%)	513 (17%)
CCI ≥ 5 (%)	411 (14%)
Any blood-pressure medication *n* (%)	1658 (55%)^e^
Formal education after upper secondary school *n* (%)	965 (33%)^f^

Abbreviations: CCI, Charlson Comorbidity Index; LISA, Longitudinal Integrated Database for Health Insurance and Labour Market Studies.

Missing data from the Riksstroke registry for a, b and d of 1%; c 2% and e and f > 1% missing data from the LISA registry, while no missing data for other variables.

### Overall rehabilitation utilisation over 24 months

A total of 42% received rehabilitation in the studied settings between discharge and 3 months after stroke, 42% during months 4–12 and 32% during months 13–24. Overall, about two-third (65%) received any rehabilitation consultation from discharge up to 3 years.

A total of 13,656 (33%) consultations or inpatient rehabilitation days occurred during the first 3 months over all settings while 17,942 (44%) consultations occurred during months 4–12, and 9632 (23%) during months 13–24. From discharge to 3 months, 16% received 1 or 2 and 6% received 3–5 consultations. Twenty percent received more than 5 consultations, accounting for 91% (12,418) of the total number of consultations during the initial 3 months. The number of consultations during months 4–12 and 13–24 was similar, see Supplementary material.

### Rehabilitation utilisation by age, sex and living situation

Rehabilitation after a stroke differed significantly between age groups (*P* ≤ .001), sexes (*P* = .001) and living situations (*P* ≤ .001) from discharge to 3 months after stroke (Table 2). The differences were most substantial between age groups; 57% of people below 65 years and 19% of those above 84 years received rehabilitation. The differences remained significant but were less pronounced during months 4–12 (*P* ≤ .001) and months 13–24 (*P* ≤ .001) for age and living situation while sex differences were non-significant at 4–12 months (*P* = .213) and 13–24 months (*P* = .120). Graphical presentation and number of consultations by age group are presented in Supplementary material in Graph S1 and Table S1.

**Table 2:** Rehabilitation utilisation at 1–3, 4–12 and 13–24 months after stroke, by age, sex and living situation.

Group	Utilisation discharge to 3 months (*n*: 2781)	*P*-value	Utilisation 4–12 months (*n*: 2678)	*P*-value	Utilisation 13–24 months (*n*: 2542)	*P*-value
**Age group *n* (%)**						
<65 years	423/739 (57%)	<.001^a^	385/727 (53%)	<.001^a^	283/708 (40%)	<.001^a^
65–74 years	375/849 (44%)		367/821 (45%)		260/788 (33%)	
75–84 years	300/841 (36%)		320/815 (39%)		228/775 (30%)	
>84 years	68/352 (19%)		62/315 (20%)		54/271 (20%)	
**Sex *n* (%)**						
Male	714/1600 (45%)	.001^a^	667/1538 (43%)	.213^a^	456/1461 (31%)	.12^a^
Female	452/1181 (38%)		467/1140 (41%)		369/1081 (34%)	
**Living situation *n* (%)** ^b^						
Single living	371/1032 (36%)	<.001^a^	376/997 (39%)	<.001^a^	273/914 (30%)	.041^a^
Living with others	783/1713 (46%)		747/1667 (45%)		540/1596 (34%)	

^a^Chi-squared test for statistically significant differences across all groups.

^b^Missing data from the Riksstroke registry of 1%.

### Rehabilitation settings

**Figure 1:**
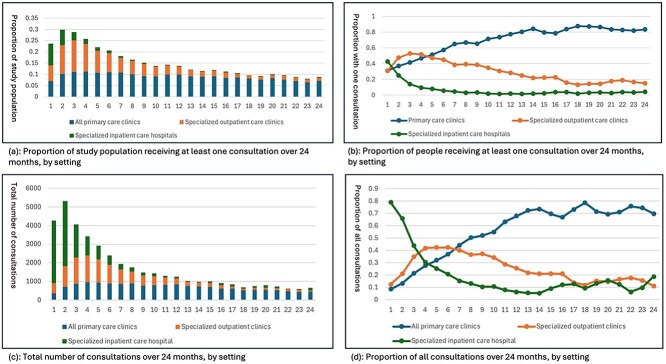
(a–d) Rehabilitation utilisation over 24 months, by setting for (a) proportion of study population who received at least one consultation; (b) proportion of with at least 1 rehabilitation consultation; (c) total number of consultations and (d) proportion of total number of consultations.

**Figure 2:**
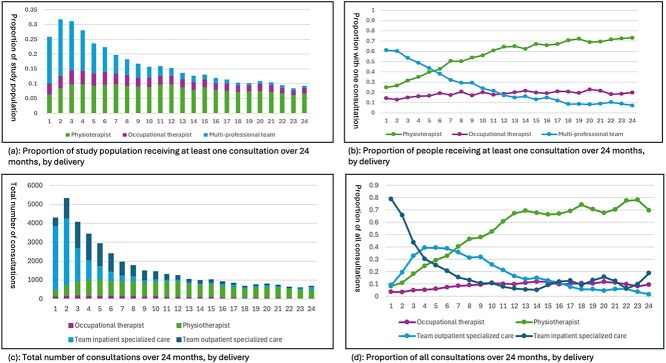
(a–d) Rehabilitation utilisation over 24 months, by delivery for (a) proportion of study population who received at least 1 consultation; (b) proportion of people with at least 1 rehabilitation consultation; (c) total number of consultations and (d) proportion of all rehabilitation consultations.

Specialised inpatient hospitals delivered rehabilitation to 10% of the study population during the first month after discharge while outpatient specialised hospital clinics delivered rehabilitation to 13% in the second and 14% in the third month after stroke (Figure 1a). Regional primary care or private clinics delivered rehabilitation to 11% in the fifth and 7% in the 24th month after stroke. For people receiving rehabilitation, rehabilitation in specialised inpatient hospitals was most common the first month after discharge (43%) and least common the following months (Figure 1b). Specialised outpatient hospital clinics were the most common setting from the second (48%) to fourth (52%) month after stroke, while primary care clinics were most common from the fifth month (51%) (Figure 2b).

Out of all rehabilitation consultations or inpatient days delivered over 24 months, 16,634 (40%) were delivered in primary care clinics, 11,611 (28%) in specialised outpatient care clinics and 12,979 (32%) rehabilitation days in specialised inpatient hospitals but this varied substantially over the months (Figure 1c). Among all rehabilitation consultations, specialised inpatient hospitalisation was the most common setting from the first (79%) to third (44%) month after stroke. Specialised outpatient clinics were most common from the fourth (42%) to sixth (42%) month, while primary care was most common from the seventh month (44%) (Figure 1d).

### Rehabilitation delivery

After discharge, multi-professional teams delivered rehabilitation to 16% of the study population during the first month and 19% during the second month, before decreasing in the following months. Physiotherapists delivered rehabilitation to 10% in the third month and 7% in the 24th month, while occupational therapists delivered rehabilitation to 5% in the third month and 2% in the 24th month (Figure 2a). For the study population receiving rehabilitation, delivery by a physiotherapist increased from the first month (25%) to the 24th month (73%), while the delivery by a multi-professional team decreased from the first month (61%) to the 24th month (7%) and delivery by an occupational therapist had less variation over time (ranging from 13% at the second month to 22% at the 20th month) (Figure 2b). No delivery by multi-professional teams was registered within regional- or private primary care clinics, but rehabilitation delivered by both a physiotherapist and an occupational therapist within the same month occurred in 665 instances (12%) over 24 months.

Out of all rehabilitation consultations over 24 months, 22,587 (55%) were delivered by multi-professional teams, 15,640 (38%) were delivered by physiotherapists and 2997 (7%) were delivered by occupational therapists (Figure 2c). The most common delivery was made by multi-professional teams from the first until the seventh month (51%), which were primarily delivered within specialised inpatient hospitals for the first (79%) and second (66%) months, while specialised outpatient clinics were more common from the fourth (39%) to seventh (33%) month. Delivery by physiotherapists was most common from the eighth (47%) to the 24th month (70%), while delivery by occupational therapists varied between the second (3%) and 14th month (12%) (Figure 2d).

## Discussion

### Summary of findings

Based on register data from a major healthcare region in Sweden, our study is the first that in detail, and over several years describes rehabilitation utilisation, delivery and settings for a general stroke population. The most prominent finding is that most people after stroke did not receive any rehabilitation consultation within the first 3 months after discharge, or up to 24 months. Still, more than 41,000 rehabilitation consultations were delivered, although to a subset of the study population and most commonly during the first months after stroke. Younger people and those living with others consistently received rehabilitation more often and frequently than older people. Furthermore, our findings show that only a small proportion of people after stroke receive multifaceted and complex interventions delivered by multi-professional teams within specialised hospital-based settings, and none within regional primary care—despite previous studies reporting multiple needs and problems after a stroke.

### Findings in relation to other studies

Compared with registry-based data from the United States, we found lower rehabilitation utilisation in the first month after discharge (23% vs 41%).^22^ Nevertheless, overall trends align with earlier findings showing that inpatient rehabilitation dominates early after stroke,^23^ physiotherapy is more common than occupational therapy,^14^ and rehabilitation utilisation varies by setting and population subgroup.^24^ A recent review^25^ explored differences in utilisation based on age and found a large variation between studies. These differences indicate a variation in utilisation that could be due to context, methodological considerations in previous studies or access to reliable healthcare utilisation data across settings.

Our findings regarding the extent of rehabilitation utilisation can be put in contrast to previous studies identifying substantial unmet needs after stroke, where most people report multiple health problems,^26^ and a vast majority report any (88%) or significant (59%) symptoms^27^ at 6 months. As information about needs at discharge was not available in our study, no direct comparisons of whether rehabilitation reaches all those with needs were possible. Still, occurring subjective experiences of inadequacy of care among people after stroke^28^ and at discharge^11^ are common.

The more intensive and prevalent rehabilitation early in the recovery process that we identified could signal greater perceived needs for rehabilitation and an increased possibility to impact any disability early after stroke.^29^^,^^30^ Our finding of limited specialised team consultations in the long term might reflect that stroke rehabilitation historically has been delivered mainly in hospital settings^31^ and not in primary health care. Guidelines recommend integrated and continuous rehabilitation,^9^^,^^12^^,^^13^ that targets a variety of causes of disability after a stroke, as well as follow-up reviews for all people after stroke.^9^ These recommendations do not align with the utilisation observed in the present study.

A previous study^32^ showed that about one-fourth of people after stroke have difficulties with activities of daily living and one-third have difficulties with walking or moving safely at 3 months post-stroke. These activity limitations may partly be explained by the current results showing a low overall utilisation of rehabilitation delivered by physiotherapists and even lower by occupational therapists beyond the initial months, which could indicate that rehabilitation practices do not meet these needs. Our results show that consultations delivered by physiotherapists are more prevalent, often targeting aspects of disabilities prioritised by people after stroke, such as balance and walking.^33^ Interventions aimed to improve physical functioning and walking—often delivered by physiotherapists—have been shown to improve outcomes.^34^ As physiotherapists constitute a larger available work force than occupational therapists in regional and private primary care in Sweden, this may explain the higher amount of consultations in the settings included in the present study.

### Strengths and limitations

This study has several strengths.

1. Includes a large general cohort of people after a first-ever stroke and is the largest and most detailed study to date exploring rehabilitation utilisation, delivery and settings with a 2-year follow-up.
2. Draws upon detailed and reliable Swedish National Stroke Registry data, healthcare utilisation data and population registries.
3. The linkage of registries through available personal identification numbers allows for the possibility to gain a more comprehensive and detailed picture of the population and the rehabilitation after stroke through multiple sources.

Several methodological limitations must be acknowledged.

1. Severity, functioning, disability, rehabilitation satisfaction and needs, assessed with tools such as the mRS^35^ and NIHSS^36^ were not available in the registries for our cohort and over our study period at discharge. Thus, this precludes subgroup analyses exploring associations of rehabilitation utilisation and needs, as well as conclusions regarding whether rehabilitation reached people with the greatest needs after discharge. Registries based on clinical practice data should aim to collect comprehensive data for these aspects to allow for further studies linking rehabilitation use to distinct needs for rehabilitation.
2. Results are specific to people in southern Sweden and to rehabilitation outside of municipal primary healthcare, limiting the generalisability. Data from municipal primary health care were not included because comprehensive and reliable registry data from these settings are not currently available.^37^ This was partly mitigated by excluding people not living in their own homes.
3. Consultations provided by professionals other than physiotherapists and occupational therapists, such as speech and language therapists, were not included, as these consultations need additional considerations related to data availability to be consistently captured. If our definitions were to expand to further professions, it would probably result in a slightly larger estimate of the total utilisation in our cohort.
4. Multi-professional team consultations should, per definition and design, include rehabilitation professionals, but we had no data to verify this assumption.
5. Rehabilitation consultations unrelated to stroke may have been included in the data from the primary care settings, particularly in later stages after stroke.

### Future research directions

The rehabilitation utilisation in Sweden highlighted in our study raises important questions for future research related to whether existing rehabilitation delivery and utilisation adequately meet the needs of people after stroke and current guidelines and policy recommendations. Additional research should explore if people with the greatest needs are reached by reliably and comprehensively measuring and including severity, functioning and needs at discharge or later in the process. Further research should also explore the content and quality of rehabilitation, the extent of rehabilitation delivered in municipal primary care and by professions other than physiotherapists and occupational therapists. Meeting short- and long-term unmet needs after stroke, and fulfilling comprehensive guideline standards, potentially require a several-fold increase in rehabilitation consultations, which may not be realistic given the available resources. Thus, health economic evaluations are important to increase the knowledge in this respect. Future development of guidelines using established systems for assessment and prioritisation^38^ could consider feasibility by balancing ideal recommendations with workforce capacity while considering whether any specific patient group should be targeted, and when rehabilitation could be best utilised.

We identified substantial overlap between different rehabilitation settings over time and considerable differences between patient groups. Further research is needed to address a rehabilitation system with notable variation in how people after stroke access, are offered, or transition between rehabilitation services, which is in line with patient experiences.^11^ Such inconsistencies raise concerns regarding equality and equity in access to rehabilitation,^39^ and warrant further attention.

## Conclusions

Less than half of the population who had a first-ever stroke in southern Sweden utilise rehabilitation provided by regional and private clinics and hospitals up to 24 months after discharge. Intensity, setting and delivery vary over time and between age groups. Further studies are warranted to evaluate whether the amount of rehabilitation delivered meets the needs after stroke, compare settings or countries over time, and include additional rehabilitation professions and delivery settings.

## Supplementary Material

Supplementary_Material_aakag086

## Data Availability

All data are archived according to the Swedish Act concerning the Ethical Review of Research Involving Humans. Data are not available for public sharing but may be available from the authors upon reasonable request.
