# Supplementary material for: Rehabilitation utilisation up to 24 months after stroke: a register-based cohort study in southern Sweden
Source: Eur Stroke J. 2026 Jul 24;11(7):aakag086. doi: 10.1093/esj/aakag086 (PMC13398692; doi:10.1093/esj/aakag086)

**Supplementary Material**

1. Descriptions of registries and variables
2. Individuals that passed away or moved over 24 month follow up
3. Intensity of rehabilitation by age-groups
4. Differences in rehabilitation utilization over age, sex and living situation
5. **Descriptions of registries and variables**

*The Swedish Stroke Registry (Riksstroke)* is a Swedish national quality register on stroke care and contains individual patient characteristics and outcome variables collected at the initial stroke event and during follow-ups. The coverage of stroke survivors is comprehensive with 89% correctly classified in 2019 (1). The individual data used in this study were collected between 2016 to 2020, comprising sociodemographic information; age; date of stroke onset; type of stroke; conscious level when arriving at the hospital; first-time or subsequent stroke; living situation; smoking; use of blood-pressure medication; self-rated support needs; mobility disability before stroke; and clinic-reported planned rehabilitation after discharge. Guidance for registration in the registry is available elsewhere (2).

*The Skåne Health care Registry* contains individual level data and on all delivered care in the Skåne region since 1998. The register includes data on primary care, secondary outpatient care and tertiary inpatient care, both private and public organized, but does not include primary health care delivered in the municipalities (3). Data collected included: Consultations by single rehabilitation professionals (i.e. physiotherapists, occupational therapists) multi professional teams); the setting where the consultations took place (i.e. primary care clinics, in-patient hospital days, outpatient hospital-based consultations); and all previous diagnose codes from healthcare utilization between 2011 to 2016 for estimating comorbidities and healthcare utilization prior to the stroke.

*The Longitudinal integrated database for health insurance and labour market studies (4)****,*** administered by Statistics Sweden (SCB) is updated yearly and contains data from several national population registries primarily collected from the Swedish Tax Agency, and contained 10.2 million individuals in 2019. The coverage is regarded high, covering education levels for 97.3% of the Swedish population 16-74 years old in 2017 and data on income or tax for 92.1% in 2019. The data included the highest educational level completed up until spring semester of 2020; disposable income on individual level and household level; place of birth.

References:

1. Riksstroke. Riksstroke Årsrapport 2019 [Internet]. 2019. Available from: Riksstroke_Årsrapport-2019_slutversionWEB-1.pdf

2. Rikstroke. Riksstroke Vagledning Akutskedet 2025 [Internet]. 2025 [cited 2025 Dec 9]. Available from: Vagledning-Akutskedet-2025.pdf

3. Löfvendahl S, Schelin MEC, Jöud A. The value of the Skåne Health-care Register: Prospectively collected individual-level data for population-based studies. Scand J Public Health. 2020 Feb;48(1):56–63.

4. Ludvigsson JF, Svedberg P, Olén O, Bruze G, Neovius M. The longitudinal integrated database for health insurance and labour market studies (LISA) and its use in medical research. Eur J Epidemiol. 2019 Apr;34(4):423–37.

1. **Individuals that passed away or moved over 24 month follow up**

**Table S1:** Study population included at each follow-up

| **Month** | **Diseased** | **Moved from county** | **People with no exclusion-event** |
| --- | --- | --- | --- |
| **0** | NA | NA | 3034 |
| **1** | *252* | *7* | 2775 |
| **2** | *50* | *2* | 2723 |
| **3** | *25* | *2* | 2696 |
| **4** | *18* | *1* | 2677 |
| **5** | *11* | *2* | 2664 |
| **6** | *19* | *3* | 2642 |
| **7** | *12* | *0* | 2630 |
| **8** | *13* | *0* | 2617 |
| **9** | *12* | *0* | 2605 |
| **10** | *12* | *1* | 2592 |
| **11** | *14* | *2* | 2576 |
| **12** | *14* | *0* | 2562 |
| **13** | *21* | *2* | 2539 |
| **14** | *13* | *2* | 2524 |
| **15** | *15* | *0* | 2509 |
| **16** | *7* | *0* | 2502 |
| **17** | *11* | *0* | 2491 |
| **18** | *5* | *2* | 2484 |
| **19** | *12* | *2* | 2470 |
| **20** | *14* | *0* | 2456 |
| **21** | *11* | *5* | 2440 |
| **22** | *11* | *1* | 2428 |
| **23** | *13* | *0* | 2415 |
| **24** | *16* | *1* | 2398 |
| Total | 590 | 35 | 2411* |
| *Included at analyses at 24 months, if either diseased or moved away took place | | | |

1. **Intensity of rehabilitation by age-groups**

**Table S2**: Rehabilitation utilization intensity for different age groups, at 1-3, 4-12 and 13-24 months.

| **Age-group** | **No rehabilitation consultations (%)** | **1–2 rehabilitation consultations (%)** | **3–5 rehabilitation consultations (%)** | **>5 rehabilitation consultations (%)** | **p-value** |
| --- | --- | --- | --- | --- | --- |
| **Utilization discharge to 3 months** | | | | | |
| <65 years | 43% | 22% | 7% | 28% | <0.001* |
| 65-74 years | 56% | 15% | 7% | 22% |  |
| 75-84 years | 65% | 15% | 5% | 16% |  |
| >84 years | 81% | 7% | 4% | 8% |  |
| **Utilization 4 to 12 months** | | | | | |
| <65 years | 47% | 14% | 7% | 31% | <0.001* |
| 65-74 years | 55% | 17% | 6% | 21% |  |
| 75-84 years | 61% | 17% | 7% | 16% |  |
| >84 years | 80% | 11% | 3% | 5% |  |
| **Utilization 13 to 24 months** | | | | | |
| <65 years | 60% | 14% | 7% | 19% | <0.001* |
| 65-74 years | 67% | 13% | 6% | 14% |  |
| 75-84 years | 71% | 13% | 5% | 12% |  |
| >84 years | 80% | 12% | 1% | 7% |  |
| *chi2-test for statistically significant differences across all groups | | | |  |  |

1. **Differences in rehabilitation utilization over age, sex and living situation**

**Graph S1**: Differences in utilization over age, sex and living situation at 1-3, 4-12 and 13-24 months.


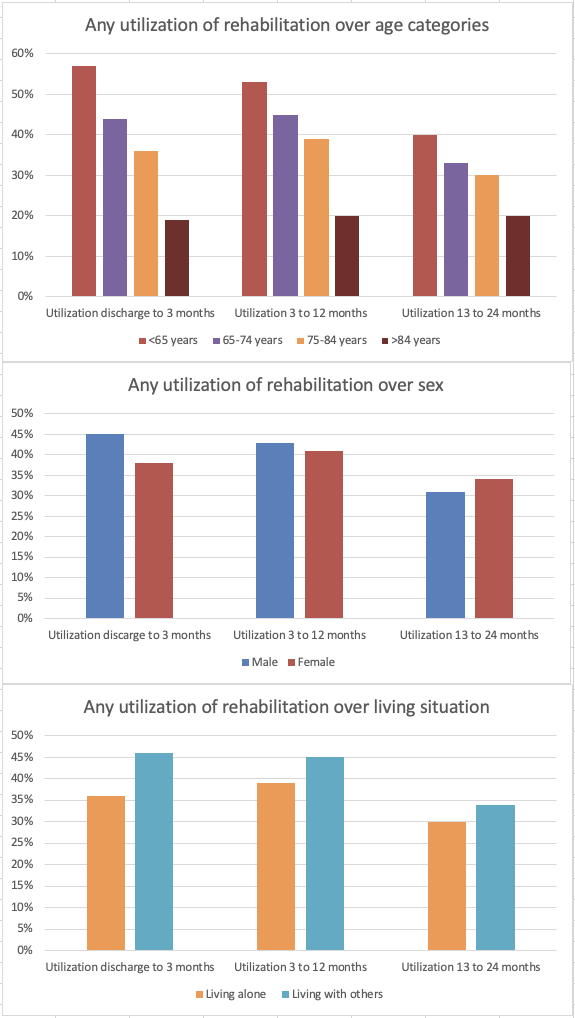

Supplement: Supplementary_Material_aakag086 [file supplementary_material_aakag086.docx]
